# Supplementary material for: A genomic approach to inferring kinship reveals limited intergenerational dispersal in the yellow fever mosquito
Source: Mol Ecol Resour. 2019 Jun 12;19(5):1254–64. doi: 10.1111/1755-0998.13043 (PMC6790672; doi:10.1111/1755-0998.13043)
Supplement: Supplementary file 1 [file MEN-19-1254-s001.pdf]

MOLECULAR ECOLOGY
RESOURCES

Supplemental Information for:

A genomic approach to inferring kinship reveals limited intergenerational dispersal in the yellow fever mosquito

Moshe Jasper*, Thomas L. Schmidt*^, Nazni W. Ahmad, Steven P. Sinkins, Ary A Hoffmann

*Equal first authors
^ Corresponding Author

Table of Contents:

**Supplementary Figure 1**	Page 2
**Supplementary Figure 2**	Page 3
**Supplementary Figure 3**	Page 4
**Supplementary Figure 4**	Page 5

MOLECULAR ECOLOGY RESOURCES

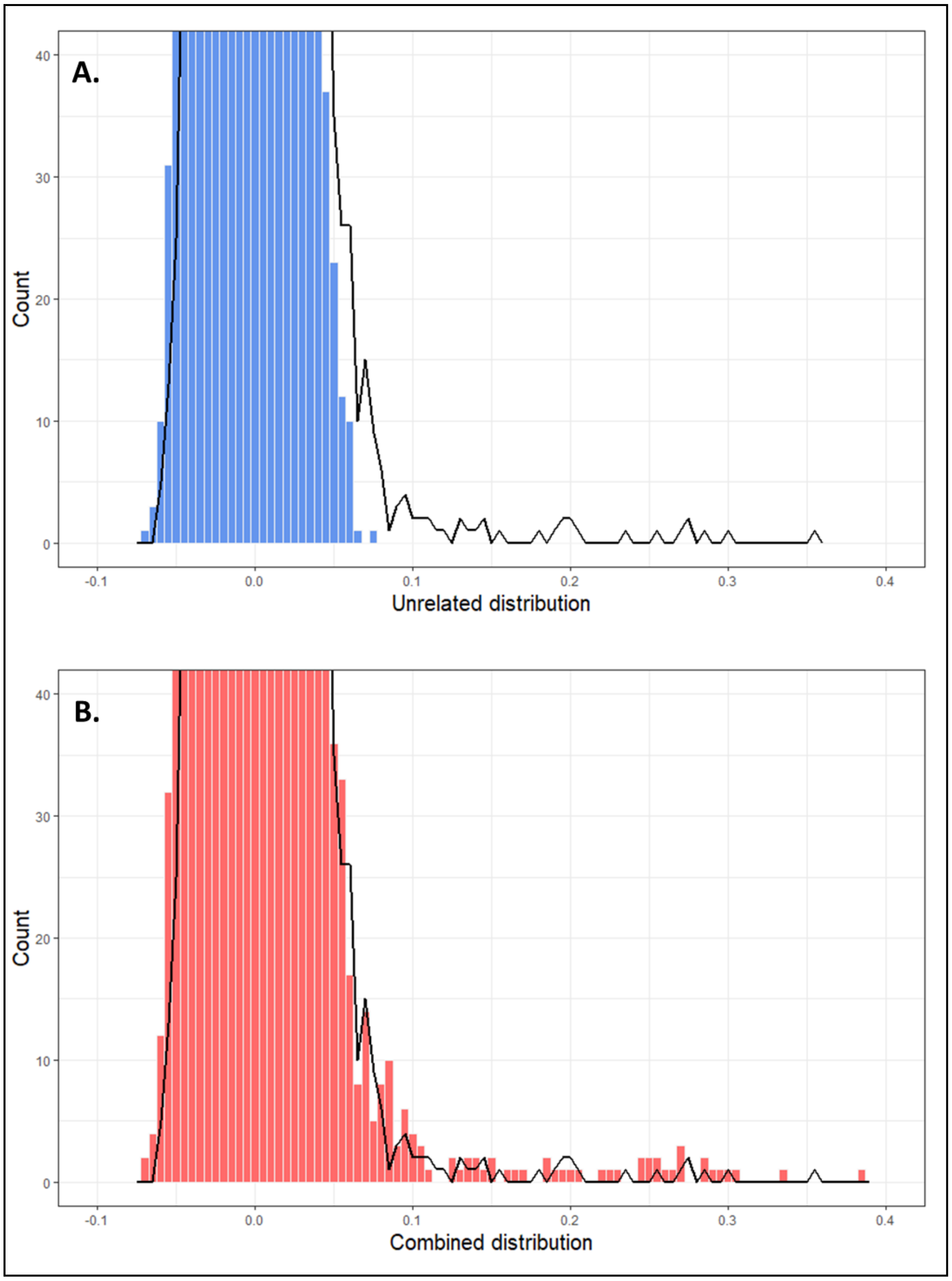

Supplementary Figure 1
A Histogram of unrelated sampling distribution (blue) compared to that of field data (black line).
B Histogram of combined sampling distribution (red) compared to that of field data (black line). The combined sampling distribution approximates the field data much more closely than does the unrelated.

MOLECULAR ECOLOGY RESOURCES

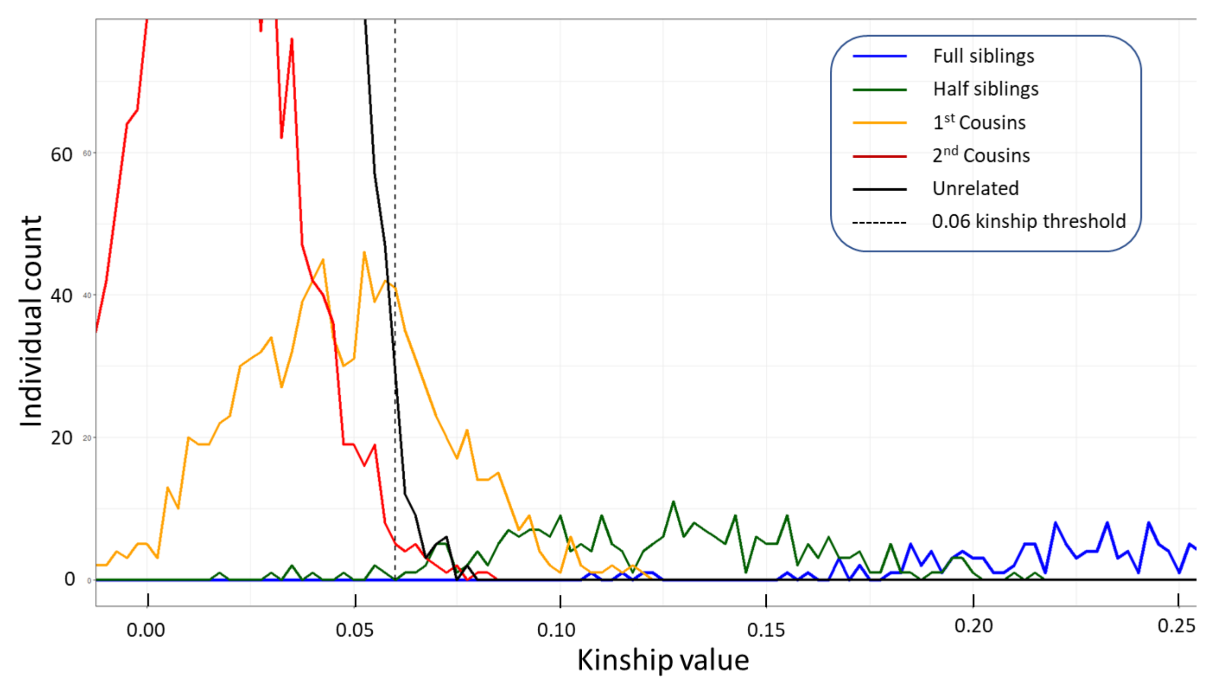

Supplementary Figure 2

Frequency polygons showing distributions of simulated kinship (*k*) scores. Colours indicate unrelated (black), second cousins (red), first cousins (orange), half-siblings (green), and full-siblings (blue). Dotted vertical line shows the *k* = 0.06 lower bound for first cousins. Based on these distributions, we no longer considered second cousins.

MOLECULAR ECOLOGY
RESOURCES

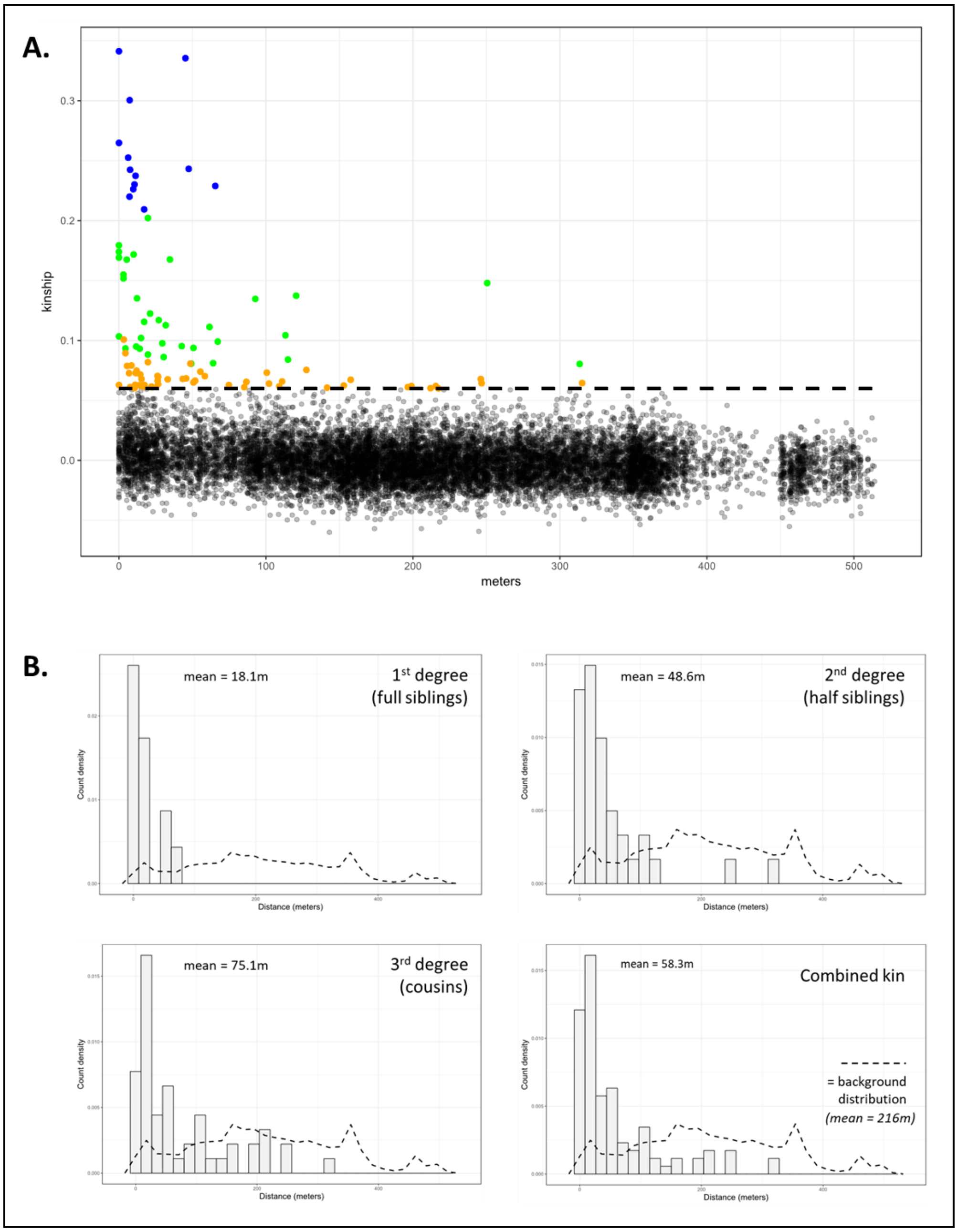

Supplementary Figure 3

Kin assignment and distribution across distance.

A Pairwise Loiselle's *k* scores between all individuals relative to distance of separation. Blue = full-siblings, green = half-siblings, orange = cousins, black = unrelated. Dotted line at $k = 0.06$ shows the lower bound for first cousins.

B Histograms of density of each order of kinship (and combined) relative to distance (m). The density distribution of all possible pairwise combinations is shown as a dotted line in each panel for reference.

MOLECULAR ECOLOGY RESOURCES

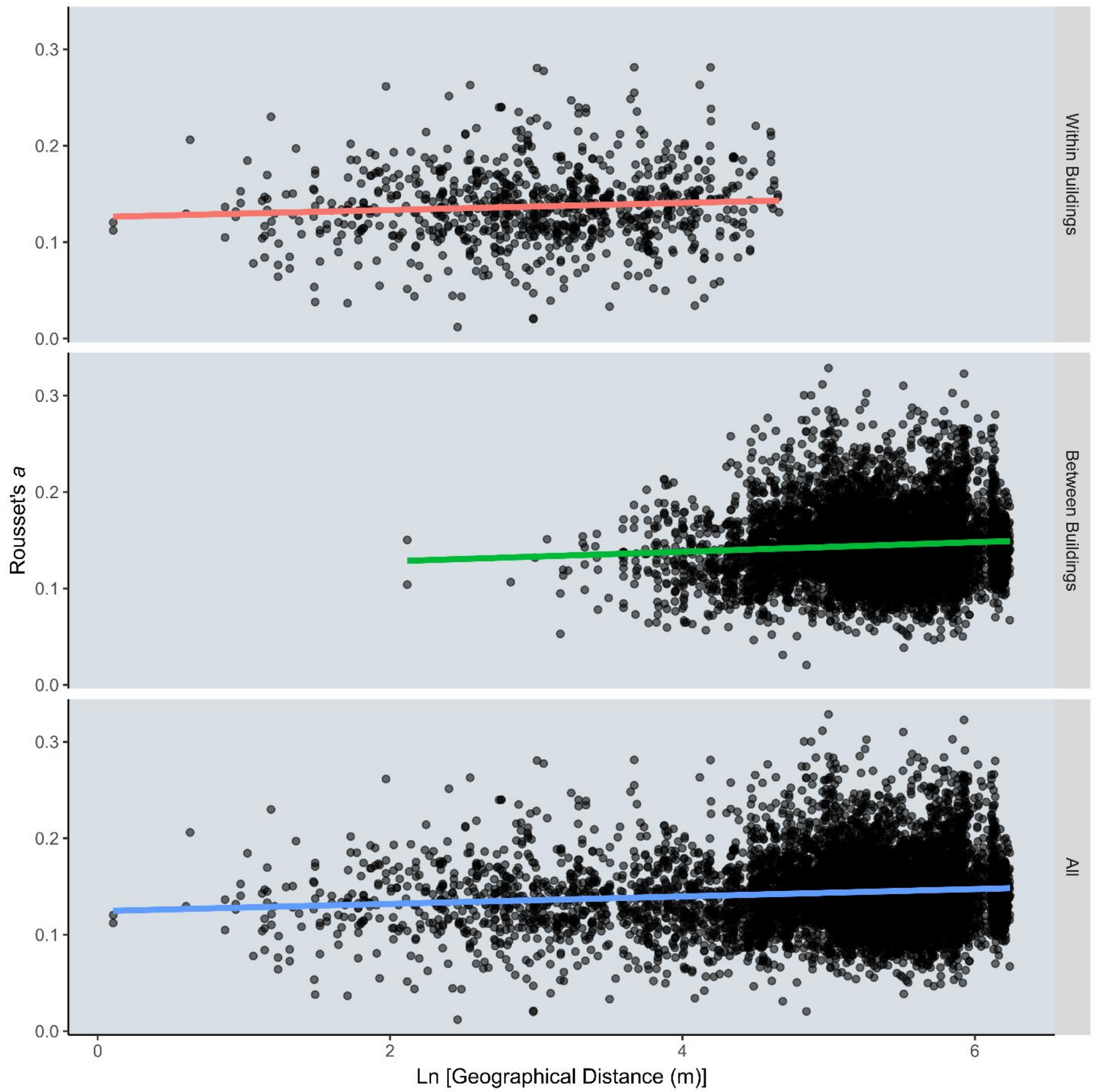

Supplementary Figure 4

Regressions of pairwise genetic distance (Rousset's *a*) and the natural logarithm of geographical distance for all non-sibling pairs. Regressions for all non-sibling pairs ($P < 2e^{-16}$; $R^2 = 0.0090$; slope = 0.0037), all non-sibling pairs within the same building ($P = 0.012$; $R^2 = 0.0062$; slope = 0.0041), and all non-sibling pairs in different buildings ($P = 1.3e^{-09}$; $R^2 = 0.0048$; slope = 0.00484) all showed significant positive associations between geographical and genetic distances.
