# Supplementary material for: A genomic approach to inferring kinship reveals limited intergenerational dispersal in the yellow fever mosquito
Source: Mol Ecol Resour. 2019 Jun 12;19(5):1254–64. doi: 10.1111/1755-0998.13043 (PMC6790672; doi:10.1111/1755-0998.13043)
Supplement: Supplementary file 2 [file MEN-19-1254-s002.pdf]

MOLECULAR ECOLOGY RESOURCES

Supplemental Information for:

A genomic approach to inferring kinship reveals limited intergenerational dispersal in the yellow fever mosquito

Moshe Jasper*, Thomas L. Schmidt*^, Nazni W. Ahmad, Steven P. Sinkins, Ary A Hoffmann

*Equal first authors
^ Corresponding Author

Calculation of Axial Standard Deviations of Dispersal

Table of Contents:

**Theory**	Page 2
**Code: Dependencies and Data Setup**	Page 3
**Code: Axial Distance Functions**	Page 4
**Final Code**	Page 6

MOLECULAR ECOLOGY RESOURCES

Theory

To enable the calculation of axial standard deviations, each dispersal process is modelled as a bivariate normal distribution with a mean of (0, 0) and rotational symmetry, allowing both axial standard deviations to be equivalent. Under these assumptions, the following procedure derives axial standard deviations from the distributions of separation distances for each kinship category:

1. For each separation distance between kin, assign a random angle of rotation. Applied aggregatively, this removes any directional sampling biases in the data while projecting the distances onto a polar coordinate system.

2. Convert the distances to one-dimensional vectors by multiplying each distance by the cosine of its rotation angle. For a polar coordinate system, this flattens the available distance information to a one-dimensional distribution centred around zero – an axial distribution.

3. Calculate the standard deviation of the resulting distribution.

4. For statistical inference by bootstrapping, apply the above steps to 1,000 subsamples produced by resampling with replacement from within the kinship category, with angles randomised in each subsample. The average of the resulting 1,000 standard deviations is an estimate of the axial standard deviation, while the 2.5% and 97.5% quantiles of the distribution define 95% confidence intervals for the estimate.

5. For Parent-Offspring inference, use equation 3 (main text) to derive an estimate from axial standard deviations of the other categories, bootstrapping on final P-O estimates.

MOLECULAR ECOLOGY RESOURCES

Code

Dependencies

The code to calculate axial deviations depends on the dplyr package
https://cran.r-project.org/web/packages/dplyr/index.html

Data setup

To run the following code, pairwise kinship and distance data must be set up as a dataframe, with each row corresponding to a separate pairwise comparison between two individuals.

A variety of fields could prove useful in this frame, but typically will at least include Loiselle's kinship (*k*), genetic distance, and geographical distance (in this example, the $meters field).

During kinship filtering, this main dataframe has been split into several smaller kinship groups (dataframes) labelled fullsibs, halfsibs, and cousins. Each dataframe contains all pairwise comparisons classified under that kinship grouping.

MOLECULAR ECOLOGY RESOURCES

Axial Distance Functions

The first function, **axialdist**, takes a distribution of distances and calculates an axial standard deviation with random angles from it (no replicates, unless specified). This corresponds to steps 1 – 3 in the paper.

```
axialdist <- function(datasource, nrep=1){
  container <- matrix(nrow = dim(datasource)[1], ncol = nrep)
  for(n in 1:ncol(container)){
    rand <- runif(dim(datasource)[1], min=0, max=2*pi)
    for(m in 1:dim(datasource)[1]) {
      container[m, n] <- datasource[m,]$meters*cos(rand[m])
    }
  }
  sd1 <- apply(container, 2, sd)
  return(sd1)
}
```

The second function, **quantax**, applies the first to 1,000 samplings of a kinship group and returns the quantiles, including those required for 95% confidence interval.

```
quantax <- function(datasource){
  return(quantile(replicate(1000, axialdist(sample_n(datasource, size=
dim(datasource)[1], rep = TRUE))[1]), c(0.025, 0.25, 0.5, 0.75, 0.975)
))
}
```

The third function, **po_quantile**, returns quantiles for parent-offspring axial standard deviation, including those required for 95% confidence intervals, through resampling the underlying distributions 1,000 times. Note that as sampling distributions for cousins overlap with those of halfsibs, a small number of trials result in Zero Division Errors, which can be discarded.

```
po_quantile <- function(fullsib_data, halfsib_data, cousin_data){
  resample_ax <- function(datasource){
    return(axialdist(sample_n(datasource, size=dim(datasource)[1], rep = TRUE
), nrep=1)[1])
  }
```

MOLECULAR ECOLOGY RESOURCES

```
  po_resample <- function(fullsib_data, halfsib_data, cousin_data){
    fullax <- resample_ax(fullsib_data)
    halfax <- resample_ax(halfsib_data)
    cousax <- resample_ax(cousin_data)
    po_ax <- sqrt(cousax**2 - 0.5*(fullax**2 + halfax**2))
    return(po_ax)
  }

  return(quantile(replicate(1000, po_resample(fullsib_data, halfsib_data, cou
sin_data)), c(0.025, 0.25, 0.5, 0.75, 0.975), na.rm = TRUE))
}
```

MOLECULAR ECOLOGY RESOURCES

Final code

Using the above data structures and functions, final axial dispersal parameters are **calculated as shown below.**

```
#axial deviations
fullsibs_axial_deviation <- mean(axialdist(fullsibs, nrep=1000))
halfsibs_axial_deviation <- mean(axialdist(halfsibs, nrep=1000))
cousins_axial_deviation <- mean(axialdist(cousins, nrep=1000))

#confidence intervals
fullsibs_quantile <- quantax(fullsibs)
halfsibs_quantile <- quantax(halfsibs)
cousins_quantile <- quantax(cousins)

#parent-offspring axial deviation
parent_offspring_axial_deviation <- sqrt(cousins_axial_deviation**2 -0.5*(fullsibs_axial_deviation**2 + halfsibs_axial_deviation**2))

#confidence interval for parent-offspring axial deviation
parent_offspring_ci <- po_quantile(fullsibs, halfsibs, cousins)
```
